# Supplementary material for: Observation of non-Hermitian topological Anderson insulator in quantum dynamics
Source: Nat Commun. 2022 Jun 9;13:3229. doi: 10.1038/s41467-022-30938-9 (PMC9184487; doi:10.1038/s41467-022-30938-9)
Supplement: Supplementary file 1 — Supplementary Information [file 41467_2022_30938_MOESM1_ESM.pdf]

Supplemental Material for "Observation of non-Hermitian topological Anderson insulator in quantum dynamics"

In this Supplemental Material, we provide details on the theoretical characterization of U and its corresponding effective Hamiltonian, as well as additional experimental data.

The effective Hamiltonian

we derive a formal expression of the effective Hamiltonian $H_{\text{eff}}(k)$, starting with the Floquet operator U in momentum space,

$$U(k) = d_0\sigma_0 + id_x\sigma_x + id_y\sigma_y + id_z\sigma_z, \tag{S1}$$

where

$$\begin{aligned} d_0 =& \cosh 2\gamma \cos 2k \cos\theta_1 \cos 2\theta_2 - \sin\theta_1 \sin 2\theta_2 + i \sinh 2\gamma \sin 2k \cos\theta_1 \cos 2\theta_2, && \text{(S2)}\\ d_x =& 0, && \text{(S3)}\\ d_y =& -\cosh 2\gamma \cos 2k \cos\theta_1 \sin 2\theta_2 - \sin\theta_1 \cos 2\theta_2 - i \sinh 2\gamma \sin 2k \cos\theta_1 \sin 2\theta_2, && \text{(S4)}\\ d_z =& \cosh 2\gamma \sin 2k \cos\theta_1 + i \sinh 2\gamma \cos 2k \cos\theta_1. && \text{(S5)} \end{aligned}$$

The effective Hamiltonian $H_{\text{eff}}(k)$ is defined by $H_{\text{eff}}(k) = i\ln U(k)$. Note the right- and left-eigenvectors of H_{eff} is the same as those of $U(k)$, which are defined as $U(k)|\psi_\pm(k)\rangle = \lambda(k)|\psi_\pm(k)\rangle$ and $U^\dagger(k)|\chi_\pm(k)\rangle = \lambda^*_\pm(k)|\chi_\pm(k)\rangle$. Here $\lambda_\pm(k) = d_0 \pm D$, where $D = i\sqrt{d_x^2 + d_y^2 + d_z^2}$. We also have

$$\begin{aligned} |\psi_\pm(k)\rangle =& \frac{1}{\sqrt{-2D^2 \pm 2id_zD}} \begin{pmatrix} id_x + d_y \\ \pm D - id_z \end{pmatrix}, && \text{(S6)}\\ |\chi_\pm(k)\rangle =& \frac{1}{\sqrt{-2(D^*)^2 \pm 2id_z^*D^*}} \begin{pmatrix} id_x^* + d_y^* \\ \mp D^* - id_z^* \end{pmatrix}. && \text{(S7)} \end{aligned}$$

It follows that $H_{\text{eff}}(k)$ can be written as

$$H_{\text{eff}}(k) = i\Big[\ln(\lambda_+(k))\frac{|\psi_+(k)\rangle\langle\chi_+(k)|}{\langle\chi_+(k)|\psi_+(k)\rangle} + \ln(\lambda_-(k))\frac{|\psi_-(k)\rangle\langle\chi_-(k)|}{\langle\chi_-(k)|\psi_-(k)\rangle}\Big]. \tag{S8}$$

Note Eq. (S8) is quite complicated, and difficult to be arranged into a tight-binding model in the coordinate space. Nevertheless, the engineering of the non-Hermitian topological Anderson insulator can be intuitively understood as introducing disorder [in the $R(\theta_1)$ operator] on top of a Floquet operator featuring non-Hermitian topology and skin effect, similar to the picture in previous theoretical proposals [S1, S2].

The generalized Brillouin zone

We briefly outline the calculation of the generalized Brillouin zone (GBZ), which is useful for later discussions. We first rewrite the Floquet operator

$$U = \sum_x |x\rangle\langle x+2| \otimes A_1 + |x+2\rangle\langle x| \otimes A_2 + |x\rangle\langle x| \otimes A_3, \tag{S9}$$

where

$$\begin{aligned} A_1 =& R(\theta_2)P_0MR(\theta_1)MP_0R(\theta_2), && \text{(S10)}\\ A_2 =& R(\theta_2)P_1MR(\theta_1)MP_1R(\theta_2), && \text{(S11)}\\ A_3 =& R(\theta_2)P_0MR(\theta_1)MP_1R(\theta_2) + R(\theta_2)P_1MR(\theta_1)MP_0R(\theta_2). && \text{(S12)} \end{aligned}$$

Here $P_0 = |0\rangle\langle 0|$ and $P_1 = |1\rangle\langle 1|$ are projectors. Note that for convenience, we denote the polarization state $|H\rangle$ ($|V\rangle$) as $|0\rangle$ ($|1\rangle$) throughout the Supplemental Material.

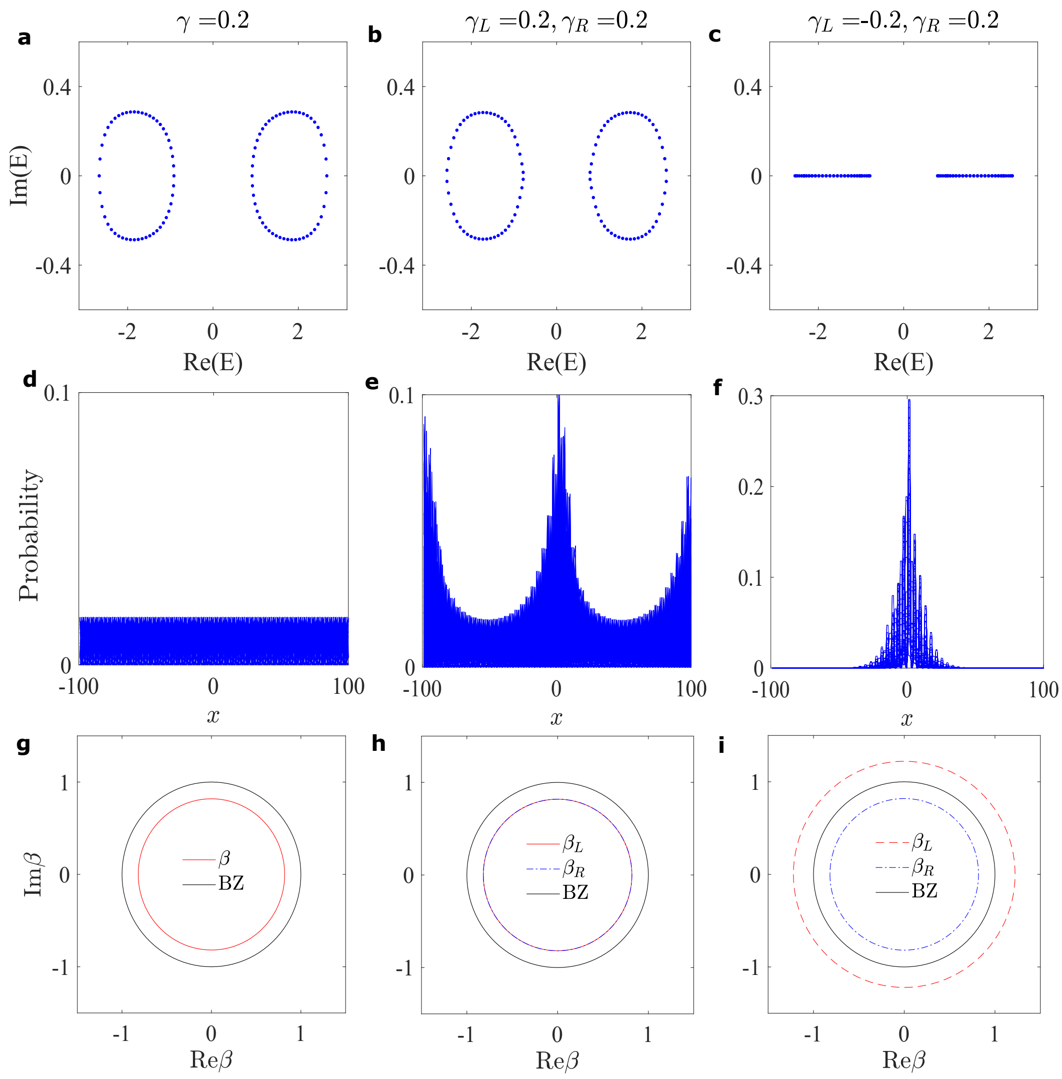

FIG. S1. Demonstration of the non-Hermitian skin effect. We take a domain-wall configuration with different parameters for $x < 0$ and $x > 0$. Therefore, two boundaries exist at $x = 0$ and $x = 100$. We show the quasienergy spectra (top panel), the spatial distribution of all eigen wavefunctions (middle panel), and the GBZs on the complex plane (lower panel). **a**, **d**, **g**, Periodic boundary condition with $\gamma = 0.2$, $\theta_1 = \pi/4$ and $\theta_2 = 0.1$. **b**, **e**, **h**, Domain-wall configuration with $\theta_1^L = \pi/4$, $\theta_2^L = 0.1$, $\theta_1^R = \pi/4$, $\theta_2^R = \pi/2$, and $\gamma_L = \gamma_R = 0.2$. The subscripts here indicate the left (L) and right (R) regions, respectively. **c**, **f**, **i**, Domain-wall configuration with the same coin parameters as those in **b**, **e** and **h**, but different loss parameters $\gamma_L = -0.2$, $\gamma_R = 0.2$. For all calculations, we take the system size of $N_c = 200$.

Following Refs. [S3, S4], we write the bulk-state ansatz as $|\psi\rangle = \sum_x \beta^x |x\rangle \otimes |\phi\rangle$. From the eigen equation $U|\psi\rangle = \lambda|\psi\rangle$ (λ is the eigenvalue), we have

$$\beta^4 + C(\lambda, \gamma, \theta_1, \theta_2)\beta^2 + e^{-4\gamma} = 0, \tag{S13}$$

where $C(\lambda, \gamma, \theta_1, \theta_2)$ is a c-number. Sorting the solutions as $|\beta_1| \le |\beta_2| \le |\beta_3| \le |\beta_4|$, and requiring $|\beta_2| = |\beta_3|$, we get

$$|\beta| = e^{-\gamma}. \tag{S14}$$

Hence, the GBZ in this case is always circular on the complex plane, with its radius dependent on γ.

Non-Hermitian skin effects of the quantum walk

The Floquet operator U features non-Hermitian skin effect in the presence of boundaries. This is explicitly shown in Fig. S1, where we calculate the quasienergy spectra (upper panel) and wavefunction distributions (lower panel)

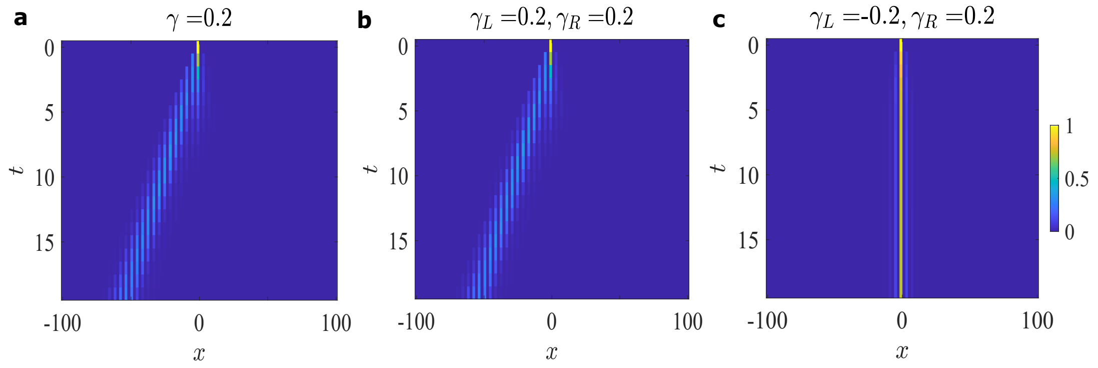

FIG. S2. Numerically simulated polarization-averaged population distribution for 20-step quantum walks near the boundary at $x = 0$ under the domain-wall configuration. The parameters for **a**, **b** and **c** respectively correspond to those in Figs. S1**a**, **b** and **c**. For the dynamics, we initialize the walker in states $|-1\rangle \otimes |0\rangle$ and $|-1\rangle \otimes |1\rangle$, respectively, independently evolve the two initial states under the same parameters, and take the average of the spatial probability distribution at each time step.

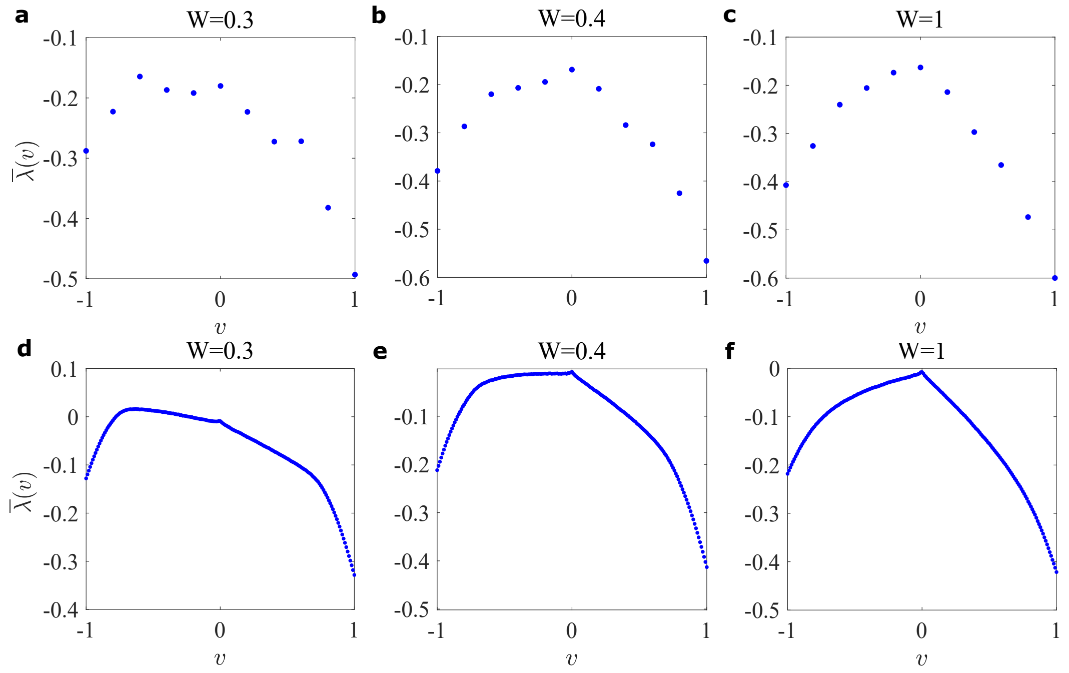

FIG. S3. Numerically calculated growth rates for quantum walks up to 10 steps in **a**, **b** and **c** and to 200 steps in **d**, **e** and **f**, respectively. We take $\theta_1 = 4.3$, $\theta_2 = 2.175$, and $\gamma = 0.1$. All data are averaged over 200 disorder configurations.

for the periodic boundary condition in **a**, **d**, **g**; a domain-wall configuration with $|\beta_L| = |\beta_R| < 1$ in **b**, **e**, **h**; and a domain-wall configuration with $|\beta_R| < 1 < |\beta_L|$ in **c**, **f**, **i**. For the domain wall configuration, the walker evolves along a ring, with $x \in [-100, 100]$ and two boundaries located at $x = 0$ and $x = 100$. The left ($x < 0$) and right ($x > 0$) regions of the ring are characterized by different parameters in **b**, **e**, **h** and **c**, **f**, **i**, respectively.

Apparently, under the periodic boundary condition, the eigen wavefunctions are not localized. Whereas under the domain-wall configuration, the wavefunctions can be localized near one of the boundaries, or both. Intriguingly, despite the non-Hermitian skin effects, the eigenspectra in Fig. S1**b** still form closed loops on the complex plane, with non-trivial spectral winding. This is in contrast to Fig. S1**c**, where no spectral winding is present. The abnormal behavior of Fig. S1**b** calls for further study in the future.

Further, in the case of Fig. S1**c**, the bulk population flows of the left and right regions are in the same direction, such that a bulk probability current persists even in the presence of boundaries. It follows that there is no dynamic population accumulation at the boundary, despite the presence of non-Hermitian skin effect. Nevertheless, dynamic population accumulation at the boundary can be observed by taking the parameters of Fig. S1**c**, where the bulk

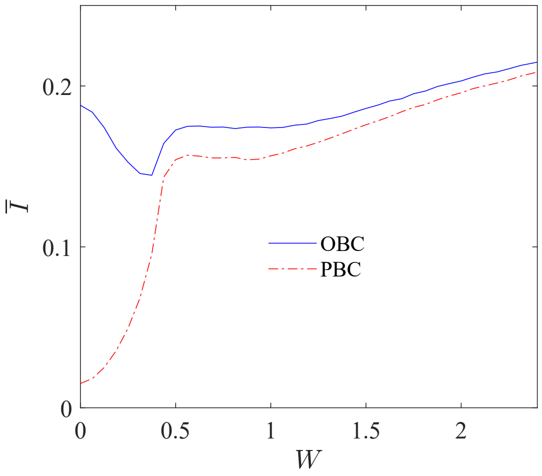

FIG. S4. Disorder-averaged IPR $\overline{I}$ as a function of the disorder strength W. We take $\theta_1 = 4.3$, $\theta_2 = 2.175$, $\gamma = 0.2$, and $N_c = 100$. The IPR is averaged over 1000 disorder configurations.

current no longer exists. In Fig. S2, we show the numerically calculated polarization-averaged probability evolution for 20-step quantum walks for these cases.

Growth rates for longer time evolutions

In Fig. S3, we show a comparison between the numerically calculated polarization-averaged growth rates for 10-step quantum walks (upper panel), and 200-step quantum walks (lower panel), respectively. The non-monotonic behavior of the growth rates with increasing W is qualitatively the same for the short and long time dynamics. This enables us to experimentally probe the competition between the two localization mechanisms using experimentally achievable time steps.

Characterizing localization through the inverse participation ratio

To provide a crosscheck on our experimental observation, we numerically calculate the inverse participation ratio (IPR) to characterize the localization properties of the system. Denoting the mth eigenstate of the Floquet operator U as $|\psi^{(m)}\rangle = \sum_{x,i} c_{x,i}^{(m)} |x,i\rangle$ $(i = H, V)$, we write the corresponding IPR as [S1]

$$I = \frac{1}{2N_c} \sum_m \sum_{i=H,V} \sum_x |c_{x,i}^{(m)}|^4, \tag{S15}$$

where N_c is the total number of unit cells (external spatial modes). In Fig. S4, we show the disorder-averaged IPR $\overline{I}$, where an additional average over different disorder configurations is performed. The calculated IPR shows a non-monotonic behavior with increasing disorder strength W, confirming the competition between the non-Hermitian skin effects and Anderson localization.

Non-Bloch topology of the quantum walk without disorder

Under the periodic boundary condition, the Floquet operator U is topological, which preserves the chiral symmetry $\Gamma U \Gamma = U^{-1}$, with $\Gamma = \mathbb{1}_w \otimes \sigma_x$. Its winding number is given by

$$\nu = \frac{1}{2\pi} \int dk \frac{-d_z \partial_k d_y + d_y \partial_k d_z}{d_y^2 + d_z^2}, \tag{S16}$$

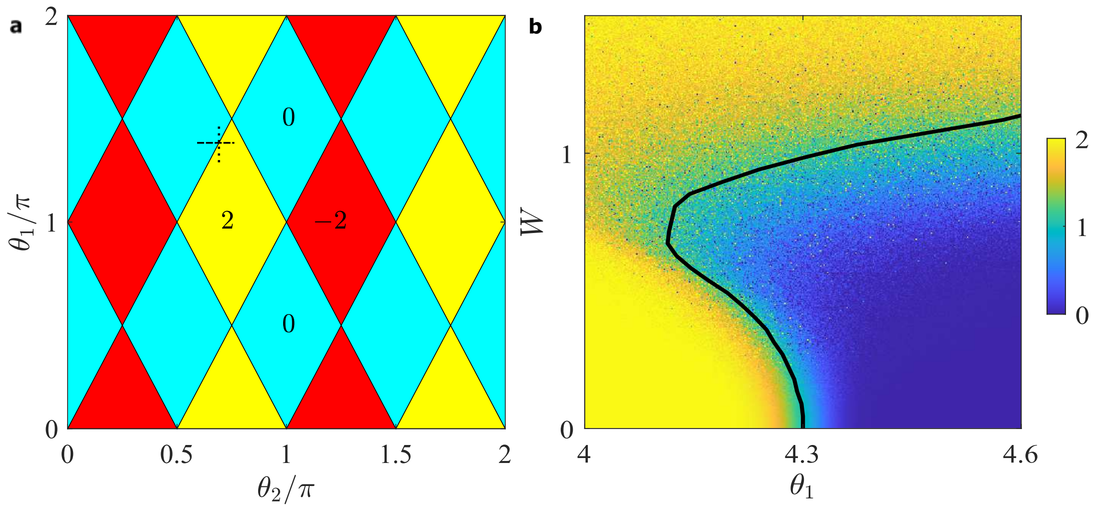

FIG. S5. **a**, Topological phase diagram in the parameter space θ_1-θ_2 characterized by the non-Bloch winding number 0, ± 2, under the open boundary condition. The thin black lines indicate the trajectory of parameter variations used in the main text. **b**, Topological phase diagram on the plane of θ_1 and W, characterized by the topological local marker. The yellow (blue) region corresponds to the topologically non-trivial (trivial) phase.

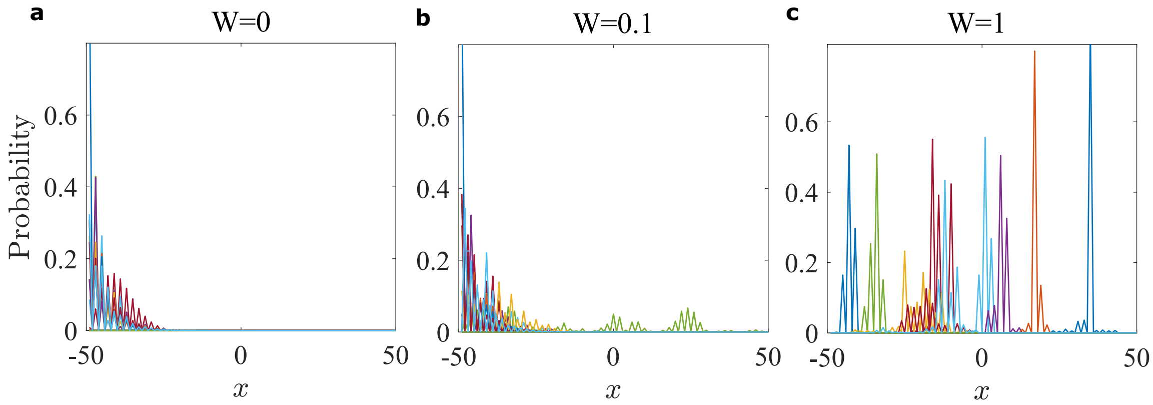

FIG. S6. Numerically evaluated spatial distribution of eigen wavefunctions under the open boundary condition, with $\theta_1 = 4.3$, $\theta_2 = 2.175$, $\gamma = 0.1$. The system size is taken to be $N_c = 100$, and the disorder strength is **a** $W = 0$, **b** $W = 0.1$, and **c** $W = 1$. Note that for visual clarity, we only plot 10 eigenstates, while the spatial distribution of all other eigenstates are qualitatively similar under a given set of parameters.

where the normalized Bloch vector $\boldsymbol{d} = \mathrm{Tr}(H\boldsymbol{\sigma})/|\mathrm{Tr}(H\boldsymbol{\sigma})|$ $[k \in [0, 2\pi)]$. Here the effective Hamiltonian H is defined through $U = e^{-iH}$, and $\boldsymbol{\sigma} = (\sigma_x, \sigma_y, \sigma_z)$ ($\sigma_{x,y,z}$ are the Pauli matrices).

Under the open boundary condition, the topology is captured by the non-Bloch winding number. For that purpose, we replace e^{ik} with $\beta = |\beta|e^{ik_\beta}$ $[k_\beta \in [0, 2\pi)]$ in Eq. (S16), and perform the integration over k_β to get the non-Bloch winding number. In Fig. S5**a**, we show the topological phase diagram in the absence of disorder. Incidentally, for our particular choice of U, the non-Bloch and Bloch phase boundaries are the same (thus independent of γ), despite the presence of the non-Hermitian skin effects and the deviation of the GBZ from the BZ.

For the completeness of discussion, we also show in Fig. S5**b** the topological phase diagram, characterized by the topological local marker, on the plane of θ_1–W. The phase diagram is qualitatively similar to that on the plane of θ_2–W in Fig. 4**a** of the main text.

Finally, to visualize the competition between disorder and the non-Hermitian skin effect, we plot in Fig. S6 typical spatial distributions of eigen wavefunctions of the system with increasing disorder strength W. In the absence of disorder (Fig. S6**a**), the non-Hermitian skin effect is reflected in the accumulation of wavefunctions at the open boundary. With a finite W, the non-Hermitian skin effect is still present, but some eigenstates start to show isolated probability peaks in the bulk, suggesting the emergence of localization (Fig. S6**b**). Finally, when W is sufficiently

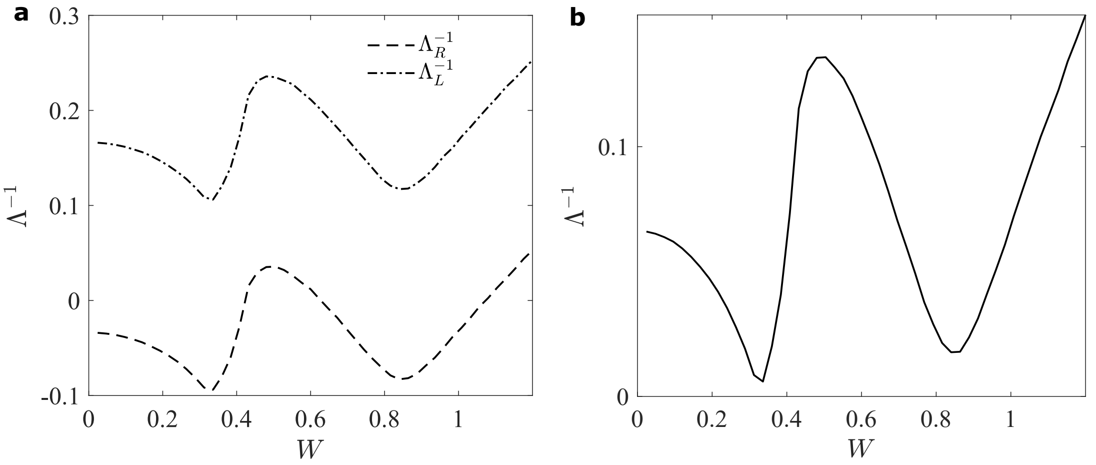

FIG. S7. Inverse localization lengths $\Lambda_{R,L}^{-1}$ in **a** and Λ^{-1} in **b** with increasing W. We take the parameters $\theta_1 = 4.3$, $\theta_2 = 2.175$, $\gamma = 0.1$, and calculate the localization length of the π-quasienergy modes ($\lambda = -1$). For our calculations, the transfer matrix is iterated over $m = 10^6$ times.

large, the non-Hermitian skin effect is completely suppressed, all eigenwavefunctions become localized in the bulk (Fig. S6**c**). Such a picture of competition is consistent with our experimentally measured growth rate in Fig. 3 of the main text.

Biorthogonal localization length

We now show how the biorthogonal localization length is calculated. As demonstrated in Fig. 4**a** of the main text, the divergence of the biorthogonal localization length overlaps the topological phase boundary, suggesting its biorthogonal criticality.

Starting from the Floquet operator U in Eq. (1) of the main text, we write $\theta_{2,x} = \theta_2$ and $\theta_{1,x} = \theta_1 + \delta_x$, where $\delta_x \in [-W, W]$ is the uniformly distributed random disorder.

Transforming U into the real space (spatial lattice modes), we have

$$\begin{aligned} U = \sum_x & A_x|x-1,0\rangle\langle x+1,0| + B_x|x-1,0\rangle\langle x+1,1| + C_x|x,0\rangle\langle x,0| + D_x|x,0\rangle\langle x,1| \\ & + E_x|x+1,0\rangle\langle x-1,0| + F_x|x+1,0\rangle\langle x-1,1| + G_x|x-1,1\rangle\langle x+1,0| + H_x|x-1,1\rangle\langle x+1,1| \\ & + I_x|x,1\rangle\langle x,0| + J_x|x,1\rangle\langle x,1| + K_x|x+1,1\rangle\langle x-1,0| + L_x|x+1,1\rangle\langle x-1,1| \end{aligned} \tag{S17}$$

with the coefficients

$$A_x = e^{2\gamma}\cos^2\theta_2\cos\theta_{1,x}, \tag{S18}$$
$$B_x = -e^{2\gamma}\cos\theta_2\sin\theta_2\cos\theta_{1,x}, \tag{S19}$$
$$C_x = -\cos\theta_2\sin\theta_2(\cos\theta_{1,x-1} + \cos\theta_{1,x+1}), \tag{S20}$$
$$D_x = \sin^2\theta_2\sin\theta_{1,x-1} - \cos^2\theta_2\sin\theta_{1,x+1}, \tag{S21}$$
$$E_x = -e^{-2\gamma}\sin^2\theta_2\cos\theta_{1,x}, \tag{S22}$$
$$F_x = -e^{-2\gamma}\sin\theta_2\cos\theta_2\cos\theta_{1,x}, \tag{S23}$$
$$G_x = e^{2\gamma}\sin\theta_2\cos\theta_2\cos\theta_{1,x}, \tag{S24}$$
$$H_x = -e^{2\gamma}\sin^2\theta_2\cos\theta_{1,x}, \tag{S25}$$
$$I_x = \cos^2\theta_2\sin\theta_{1,x-1} - \sin^2\theta_2\sin\theta_{1,x+1}, \tag{S26}$$
$$J_x = -\cos\theta_2\sin\theta_2(\sin\theta_{1,x-1} + \sin\theta_{1,x+1}), \tag{S27}$$
$$K_x = e^{-2\gamma}\cos\theta_2\sin\theta_2\cos\theta_{1,x}, \tag{S28}$$
$$L_x = e^{-2\gamma}\cos^2\theta_2\cos\theta_{1,x}. \tag{S29}$$

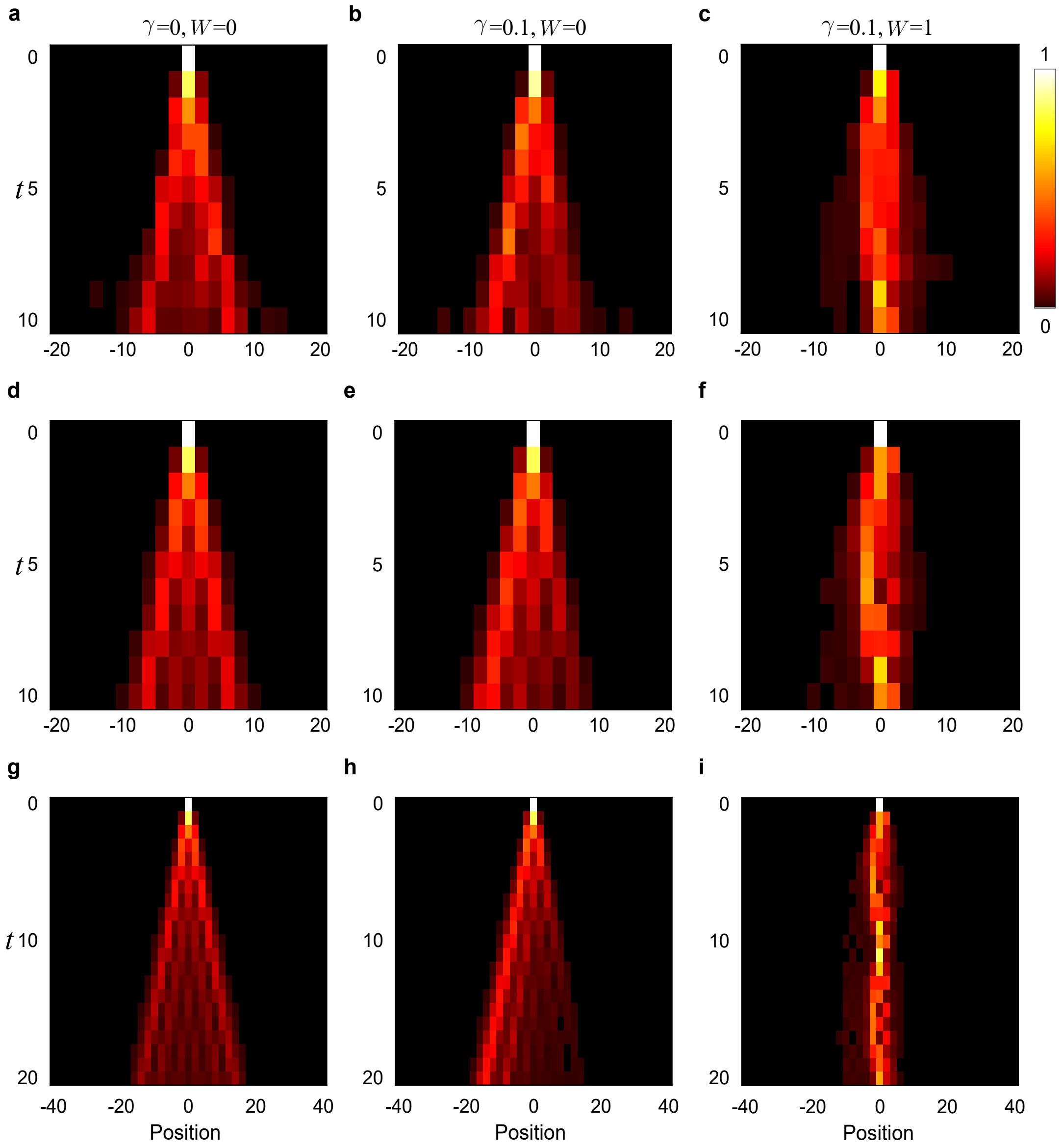

FIG. S8. **a**, **b**, **c**, Measured polarization-averaged probability distribution under different loss and disorder parameters. The probability distribution is averaged over two independent 10-step quantum walks with the initial states $|0\rangle \otimes |H\rangle$ and $|0\rangle \otimes |V\rangle$, respectively. The coin parameters are $\theta_1 = 4.3$ and $\theta_2 = 2.175$. **d**, **e**, **f**, Numerically simulated polarization-averaged probability distribution following a 10-step quantum walk, with parameters corresponding to those in **a**, **b** and **c**. **g**, **h**, **i**, Simulated polarization-averaged probability distribution following a 20-step quantum walk, with the same parameters as those of **d**, **e** and **f**. In **c**, **f**, and **i**, only one disorder configuration is taken.

Writing the right eigenstate of U as $|\psi_R\rangle = \sum_x c_{x,0}|x,0\rangle + c_{x,1}|x,1\rangle$, we have from the right eigen equation $U|\psi_R\rangle = \lambda|\psi_R\rangle$ (λ is the eigenvalue)

$$A_{x+1}c_{x+2,0} + B_{x+1}c_{x+2,1} + (C_x - \lambda)c_{x,0} + D_x c_{x,1} + E_{x-1}c_{x-2,0} + F_{x-1}c_{x-2,1} = 0, \tag{S30}$$

$$G_{x+1}c_{x+2,0} + H_{x+1}c_{x+2,1} + I_x c_{x,0} + (J_x - \lambda)c_{x,1} + K_{x-1}c_{x-2,0} + L_{x-1}c_{x-2,1} = 0. \tag{S31}$$

Noticing that $\frac{A_x}{G_x} = \frac{B_x}{H_x} = \cot\theta_2$, and $\frac{E_x}{K_x} = \frac{F_x}{L_x} = -\tan\theta_2$, we have

$$\mathcal{M}\begin{pmatrix} c_{x+2,0} \\ c_{x+2,1} \end{pmatrix} = \mathcal{N}\begin{pmatrix} c_{x,0} \\ c_{x,1} \end{pmatrix}, \tag{S32}$$

where

$$\mathcal{M}=\begin{pmatrix} C_{x+2}-\lambda-I_{x+2}\cot\theta_2 & D_{x+2}-(J_{x+2}-\lambda)\cot\theta_2 \\ G_{x+1} & H_{x+1} \end{pmatrix}, \tag{S33}$$

$$\mathcal{N}=-\begin{pmatrix} E_{x+1}-K_{x+1}\cot\theta_2 & F_{x+1}-L_{x+1}\cot\theta_2 \\ I_x\sin^2\theta_2+(C_x-\lambda)\cos\theta_2\sin\theta_2 & (J_x-\lambda)\sin^2\theta_2+D_x\cos\theta_2\sin\theta_2 \end{pmatrix}. \tag{S34}$$

We subsequently derive in an iterative fashion

$$\begin{pmatrix} c_{2m,0} \\ c_{2m,1} \end{pmatrix}=\mathcal{T}_R^m\begin{pmatrix} c_{0,0} \\ c_{0,1} \end{pmatrix}, \tag{S35}$$

where $\mathcal{T}_R=\mathcal{M}^{-1}\mathcal{N}$ is identified as the right transfer matrix.

Similarly, we start from the left eigen equation $U^\dagger|\psi_L\rangle=\lambda|\psi_L\rangle$ ($|\psi_L\rangle$ being the left eigenstate of U), and derive the left transfer matrix $\mathcal{T}_L$.

We may then define the right localization length Λ_R through $\Lambda_R^{-1}=\lim_{m\to\infty}\ln|T_{R,m}|/2n$; and the left localization length Λ_L through $\Lambda_L^{-1}=\lim_{m\to\infty}\ln|T_{L,m}|/2n$, where $T_{R,m}$ and $T_{L,m}$ are respectively the largest eigenvalues of $\mathcal{T}_R^m$ and $\mathcal{T}_L^m$. Note that, since $U^\dagger(\gamma)=\Gamma U(-\gamma)\Gamma$, and the chiral symmetry operator $\Gamma=\sum_x|x\rangle\langle x|\otimes\sigma_x$ is local, Λ_L of $U(\gamma)$ is equal to Λ_R of $U(-\gamma)$. The biorthogonal location length Λ is then defined as $\Lambda^{-1}=(\Lambda_R^{-1}+\Lambda_L^{-1})/2$.

In Fig. S7, we plot the numerically calculated inverse localization lengths $\Lambda_{R,L}^{-1}$ and Λ^{-1}. Only the biorthogonal localization length diverges at the topological phase boundary $W=0.32$ and $W=0.84$. Note that when increasing the size of the system (n), Λ^{-1} approaches zero at $W=0.32$ and $W=0.84$ in Fig. S7**b**.

	γ	W												
		0	0.1	0.2	0.3	0.4	0.5	0.6	0.7	0.8	0.9	1	1.1	1.2
1	0	2	2.217018	2.339901	0.509919	1.329784	0.22217	0.112614	0.487627	1.169285	0.097685	1.608114	0.400083	1.840983
	0.1	2	2.258153	2.105137	1.538132	0.397068	1.113619	0.093654	-0.20871	1.908419	0.691345	2.285748	1.756732	1.402385
2	0	2	1.883426	1.647853	1.626782	0.855748	-0.0988	0.008618	-0.2103	1.994606	0.926572	0.545968	0.616436	2.796825
	0.1	2	2.144383	1.864388	0.126553	0.622363	0.094496	0.093578	-0.11809	0.836388	1.874557	2.001842	1.603849	2.143547
3	0	2	2.075454	2.11795	1.668409	-0.14964	-0.02008	-0.22759	0.423964	2.17E-05	0.675517	1.390595	1.648498	2.007943
	0.1	2	1.773224	2.535687	1.446074	1.561309	0.038571	0.037713	-0.5052	0.307323	1.804518	1.711522	2.161712	2.11136
4	0	2	1.841091	2.187248	1.338127	0.790009	0.973508	-0.33957	-0.61909	1.826037	1.646009	1.581361	1.617964	1.985449
	0.1	2	2.028671	1.99922	1.276272	0.871549	-0.43584	0.004674	1.107244	1.622292	2.044418	2.05403	1.650058	2.312589
5	0	2	1.637712	2.145472	1.238708	0.951667	0.195838	0.259527	0.150226	-0.03138	1.710136	1.971194	1.192975	4.179686
	0.1	2	1.676814	1.967182	1.537047	0.529156	0.059652	0.554732	0.533175	0.424352	0.802518	0.544488	1.955504	1.812892

TABLE I. Five sets of typical raw numerical data from 200 random-disorder configurations in Fig. 4**b**.

Additional experimental data

In Fig. S8, we show the polarization-averaged probability distribution in the bulk, both from experimental measurements and numerical simulations. Comparing Figs. S8**a** and **b**, we see that under the non-Hermitian skin effect, the probability distribution tends to flow to the left (or to the right under other parameters), due to the presence of a bulk current. For sufficiently large W, the probability becomes localized for all times. These observations are consistent with those in Figs. 2 and 3 of the main text.

To complement the bulk dynamic detections in the main text, here we directly demonstrate the existence of non-Hermitian skin effects in the presence of boundaries in Fig. S9. We consider a domain-wall geometry of non-unitary quantum walks, involving two regions with fixed coin parameters $\theta_1=4$ and $\theta_2=1.63$, but different loss parameters, e.g., γ_L and γ_R for the left ($x<0$) and right ($x\geq 0$) regions, respectively. Experimentally, to create a domain wall, we use EOMs to replace HWPs, which are introduced into each path to realize loss operator $M_{\rm E}$. For $x<-1$ (in our experiment of 9-step quantum walk, we take $x\in[-14,-1]$ for the left region), the operator of the first EOM is $\tilde{R}_{\rm EOM}=\begin{pmatrix}\cos\frac{\vartheta}{2} & -i\sin\frac{\vartheta}{2}\\ i\sin\frac{\vartheta}{2} & \cos\frac{\vartheta}{2}\end{pmatrix}$, with $\cos\frac{\vartheta_1}{2}=e^{2\gamma_L}$; while the second EOM does nothing. By contrast, for $x\geq 0$ (experimentally, $x\in[0,22]$), the first EOM does nothing and the operator of the second EOM is given by $\tilde{R}_{\rm EOM}$ but with $\cos\frac{\vartheta_1}{2}=e^{-2\gamma_R}$.

For all experiments, we initialize the walker at the bulk position $x=4$ with the coin state $|V\rangle$. First, we choose $\gamma_L=\gamma_R=0.1$ and $W=0$, where there is no boundary. The probability distribution shows a directional flow (Figs. S9**a** and **d**), which is the origin of the non-Hermitian skin effect. Second, we choose $\gamma_L=\gamma_R=-0.1$ and $W=0$. The walker, initialized near $x=4$, becomes localized at the domain-wall boundary due to the non-Hermitian

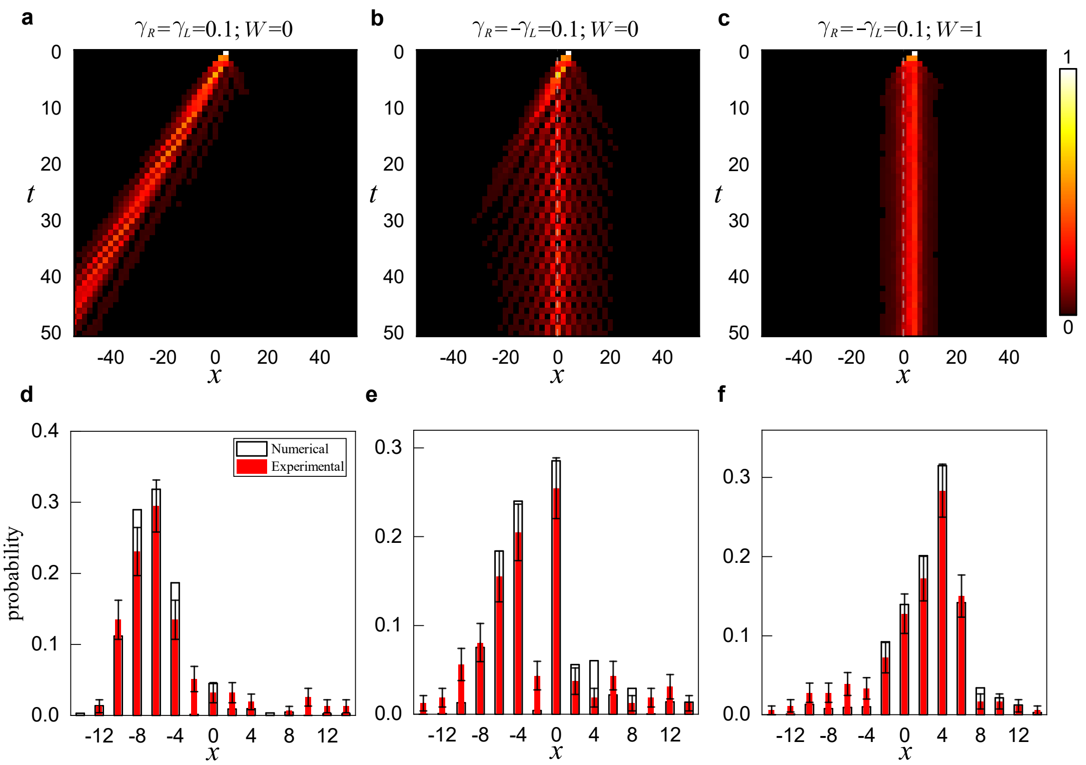

FIG. S9. Direct demonstration of the non-Hermitian skin effect near boundaries. We consider a domain-wall geometry of non-unitary quantum walks, with two regions featuring fixed coin parameters $\theta_1 = 4$ and $\theta_2 = 1.63$ but different loss parameters. Specifically, γ_L and γ_R for the left ($x < 0$) and right ($x \geq 0$) regions, respectively. The walker starts from the bulk position $x = 4$ with the initial coin state $|V\rangle$. **a**, **b**, **c**, Numerical simulations of polarization-averaged probability distribution of quantum walks under different loss and disorder parameters up to 50 steps. **d**, **e**, **f**, Measured probability distributions of 9-step quantum walks. In **c** and **f**, we average over 100 and 10 disorder configurations, respectively. The vertical dashed lines (at $x = -0.5$) in **b** and **c** indicate the boundary location.

skin effect, in sharp contrast to the first case (Figs. S9**b** and **e**). Note that since the coin parameters are the same for the two regions, the localization is driven only by the non-Hermitian skin effect but not related to the topological edge states. Finally, we choose $\gamma_L = \gamma_R = -0.1$ and $W = 1$. The probability peaks at the initial site $x = 4$ as the Anderson localization completely suppresses the probability flow in the bulk (Figs. S9**c** and **f**). This is a direct evidence for the competition between the disorder-induced Anderson localization and the non-Hermitian skin effect. The new results are consistent with our bulk measurements of the Lyapunov exponent.

In Table I, we also provide typical raw numerical data from 200 random-disorder configurations in Fig. 4**b** of the main text.

Photon loss

In our experiment, photon loss is caused by the experimental setup. Even for a unitary quantum walk, our single-loop round-trip efficiency is about 0.59. This is estimated by multiplying the efficiency of each individual element in the round trip: the collection efficiency from free space to fiber (~ 0.70), transmission rates of a beam splitter (~ 0.95), that of an EOM (~ 0.95), those of all the other optical elements (~ 0.93). We therefore have $0.59 \simeq 0.70 \times 0.95 \times 0.95 \times 0.93$.

The horizontal dashed lines in Figs. 2 and 3 indicate the event in which at most one photon (one or less one) is detected by an APD placed at some position x. Then the Lyapunov exponent becomes $\lambda_{\min}(v) = \frac{1}{t} \log \sqrt{\frac{1}{N}}$, where N is the total photon number after the t-th step. Experimentally the total photon number N is limited by the single-loop round-trip efficiency. If the values are below the dashed lines, we are not able to reconstruct them from the measured probabilities (or photon numbers). Thus, the horizontal dashed lines in Figs. 2 and 3 as the threshold values below which experimental data are no longer reliable due to photon loss.

[S1] Zhang, D.-W., Tang, L.-Z., Lang, L.-J., Yan, H. & Zhu, S.-L. Non-Hermitian topological Anderson insulators. *Sci. China-Phys. Mech. Astron.* **63**, 267062 (2020).

[S2] Luo, X.-W. & Zhang, C. Non-Hermitian disorder-induced topological insulators. arXiv: 1912.10652.

[S3] Xiao, L. *et al.* Observation of non-Hermitian bulk-boundary correspondence in quantum dynamics. *Nat. Phys.* **16**, 761 (2020).

[S4] Xiao, L. *et al.* Observation of non-Bloch parity-time symmetry and exceptional points. *Phys. Rev. Lett.* **126**, 230402 (2021).
